# Supplementary material for: FUNDC2 promotes liver tumorigenesis by inhibiting MFN1-mediated mitochondrial fusion
Source: Nat Commun. 2022 Jun 17;13:3486. doi: 10.1038/s41467-022-31187-6 (PMC9203792; doi:10.1038/s41467-022-31187-6)
Supplement: Supplementary file 1 — Supplementary Information [file 41467_2022_31187_MOESM1_ESM.pdf]

Supplementary information

FUNDC2 promotes liver tumorigenesis by inhibiting MFN1-mediated mitochondrial fusion

Shuaifeng Li, Shixun Han, Qi Zhang, Yibing Zhu, Haitao Zhang, Junli Wang, Yang Zhao, Jianhui Zhao, Lin Su, Li Li, Dawang Zhou, Cunqi Ye, Xin-Hua Feng, Tingbo Liang, Bin Zhao

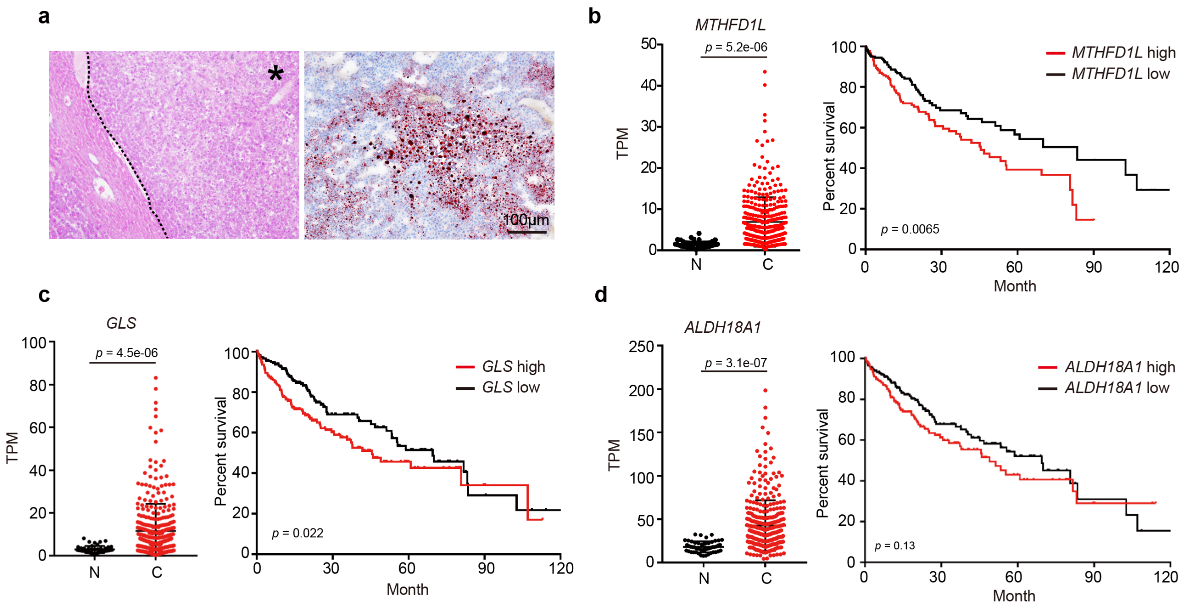

Supplementary Fig. 1 Analysis of expression and correlation with survival for genes identified in Fig. 1a. a HE and oil red staining of mouse liver tumors induced by *MYC+RAS*. Experiments were repeated five times. *MTHFD1L* (**b**), *GLS* (**c**), and *ALDH18A1* (**d**) mRNA levels in the TCGA cohort of HCC, and respective Kaplan-Meier plots of overall survival for expression levels of the specific gene. The median expression levels were used as cut-offs. Data is presented as mean ± SD. *p* values were calculated by two-tailed unpaired Student's *t*-test. Source data are provided as a Source Data file.

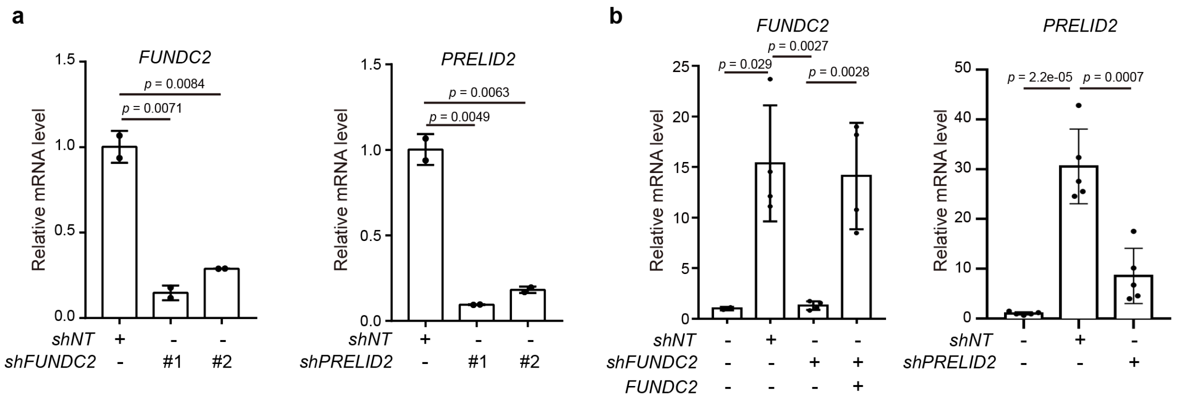

Supplementary Fig. 2 Knockdown of *FUNDC2* suppresses liver tumorigenesis. a Evaluation of shRNA knockdown efficiency in H2.35 mouse liver cell line by quantitative RT-PCR, n = 2. **b** Confirmation of *FUNDC2* and *PRELID2* knockdown and rescue in *MYC+RAS* tumors by quantitative RT-PCR, n = 5. n was biological replicates for all experiments. Data is presented as mean ± SD. *p* values were calculated by two-tailed unpaired Student's *t*-test. Source data are provided as a Source Data file.

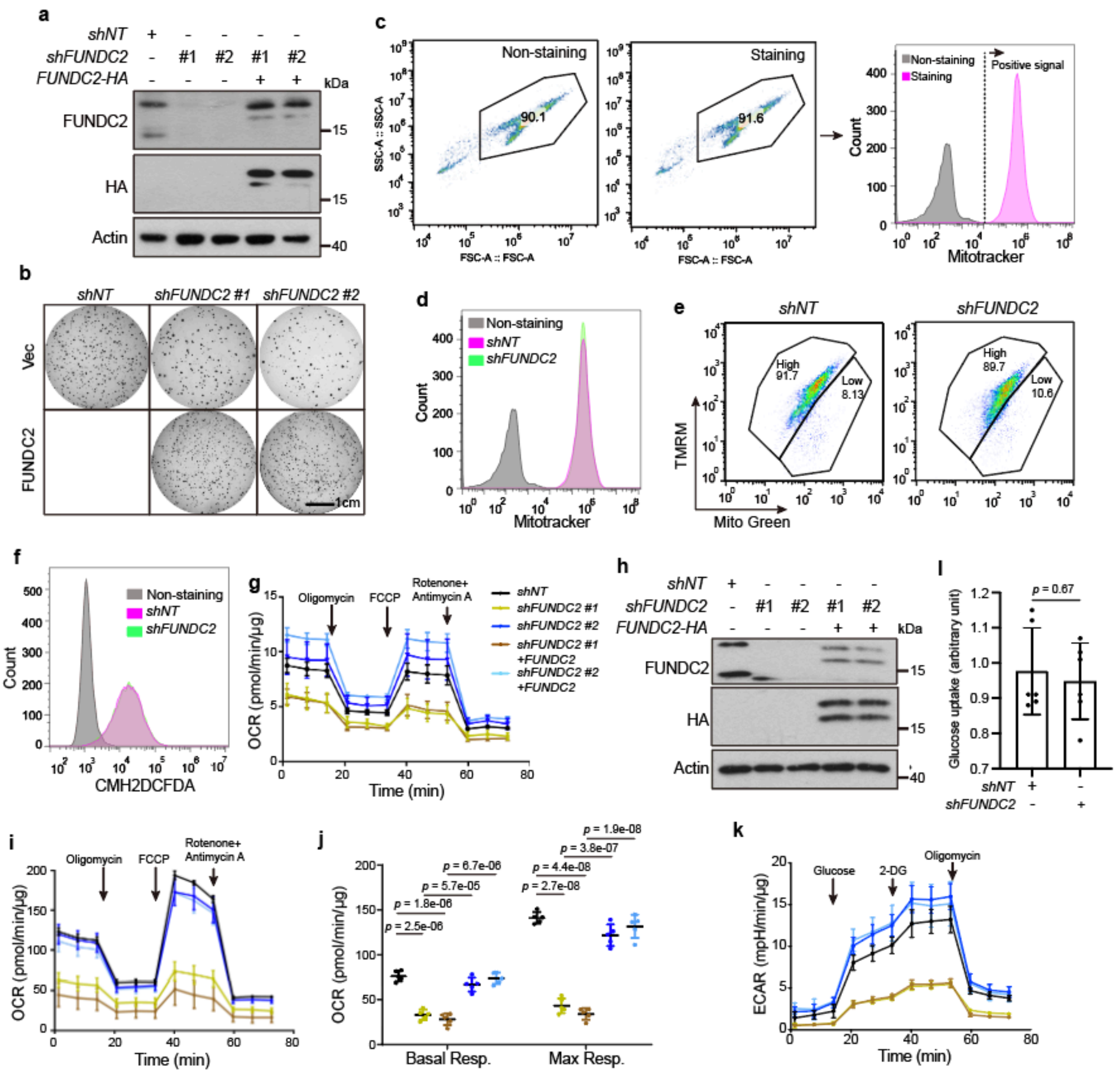

Supplementary Fig. 3 FUNDC2 promotes mitochondrial fragmentation and respiration. **a** Generation of *FUNDC2* knockdown and rescue cells. Huh-7 cells were infected as indicated, and FUNDC2 levels were determined by western blotting. **b** Knockdown of *FUNDC2* in Huh-7 cells reduced colony formation. Representative pictures of triplicates were shown. **c** Gating strategy of flow cytometry experiments. Cell populations were gated according to FSC/SSC parameters, and staining-positive populations were gated according to non-staining negative controls. **d-f** Knockdown of *FUNDC2* did not affect mitochondria mass, mitochondrial membrane potential, or ROS in Huh-7 cells. FACS analysis of cells stained by Mitotracker Green (**d**), Mitotracker Green and TMRM (**e**), and CMH2DCFDA (**f**). **g** *FUNDC2* knockdown impairs mitochondrial respiration. Indicated Huh-7 stable cells were subjected to Seahorse analysis, n = 5. **h** Generation of *FUNDC2* knockdown and rescue HepG2 cells. FUNDC2 levels were determined by western blotting. Experiments were repeated twice. **i, j** *FUNDC2* knockdown impairs mitochondrial respiration. Indicated HepG2 stable cells were subjected to Seahorse analysis. Quantification of basal and maximal respiration were showed in (**j**), n = 5. **k** *FUNDC2* knockdown impairs glycolysis. Indicated Huh-7 stable cells were subjected to Seahorse analysis, n = 5. **l**. Cellular glucose uptake was not inhibited by *FUNDC2* knockdown. Huh-7 cells were fed with 2-deoxyglucose, and its uptake was measured by a Glucose Uptake Assay Kit, n = 6. n was biological replicates for all experiments. Data is presented as mean ± SD. *p* values were calculated by two-tailed unpaired Student's *t*-test. Source data are provided as a Source Data file.

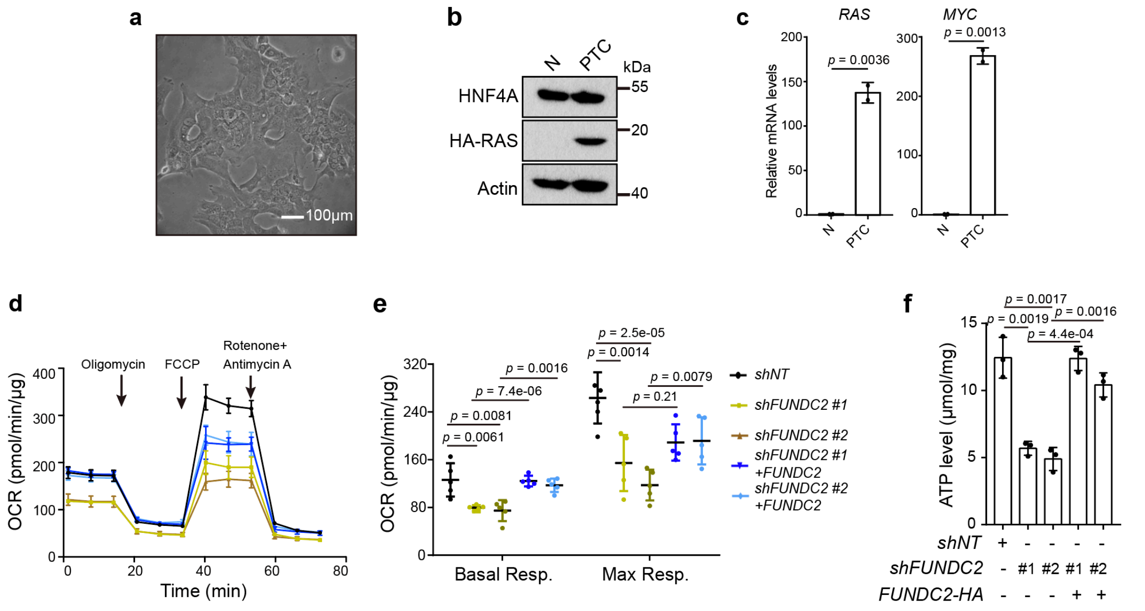

Supplementary Fig. 4 FUNDC2 promotes mitochondrial respiration in *MYC+RAS* primary tumor cells. **a** Morphology of *MYC+RAS* primary tumor cells. **b** Western blotting of RAS as a driver gene and HNF4A as a marker of hepatocytes. N, normal liver tissue; PTC, primary tumor cells. **c** Analysis of oncogene expression by quantitative RT-PCR, n = 2. **d** *FUNDC2* knockdown impairs mitochondrial respiration in *MYC+RAS* primary tumor cells. Indicated cells were subjected to Seahorse analysis, n = 5 **e** Quantification of basal and maximal respiration in (**d**), n = 5. **f** *FUNDC2* knockdown reduced cellular ATP level in *MYC+RAS* primary tumor cells, n = 3. n was biological replicates for all experiments. Data is presented as mean ± SD. *p* values were calculated by two-tailed unpaired Student's *t*-test. Source data are provided as a Source Data file.

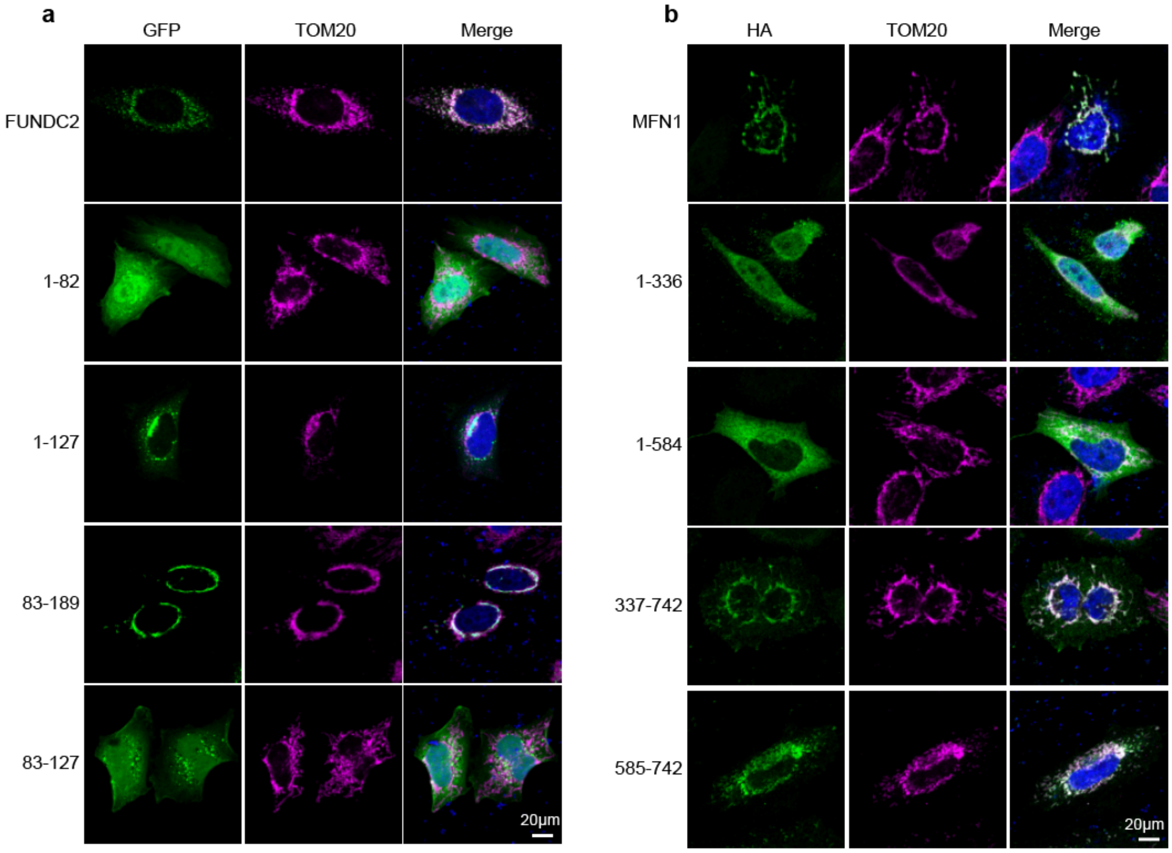

Supplementary Fig. 5 Subcellular localization of FUNDC2 and MFN1 mutants. FUNDC2 (**a**) and MFN1 (**b**) mutants were expressed in HeLa cells, and visualized by co-staining with a mitochondrial marker TOM20.

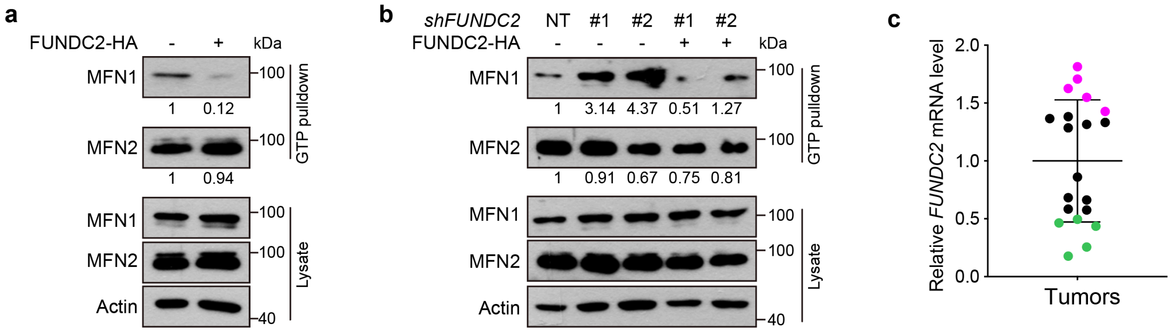

Supplementary Fig. 6 FUNDC2 inhibits GTP loading of MFN1 in HepG2 cells. *FUNDC2* was overexpressed (**a**) or knocked down (**b**) in HepG2 cells. Cell lysates were subjected to pulldown by GTP-agarose, and samples were then examined by western blotting. Quantification was done using Image J. **c** *FUNDC2* mRNA expression levels as determined by quantitative RT-PCR. Samples with highest (magenta) and lowest (green) expression were used in Fig. 5e, n = 20 tumors. Data is presented as mean ± SD with all data points shown. Source data are provided as a Source Data file.

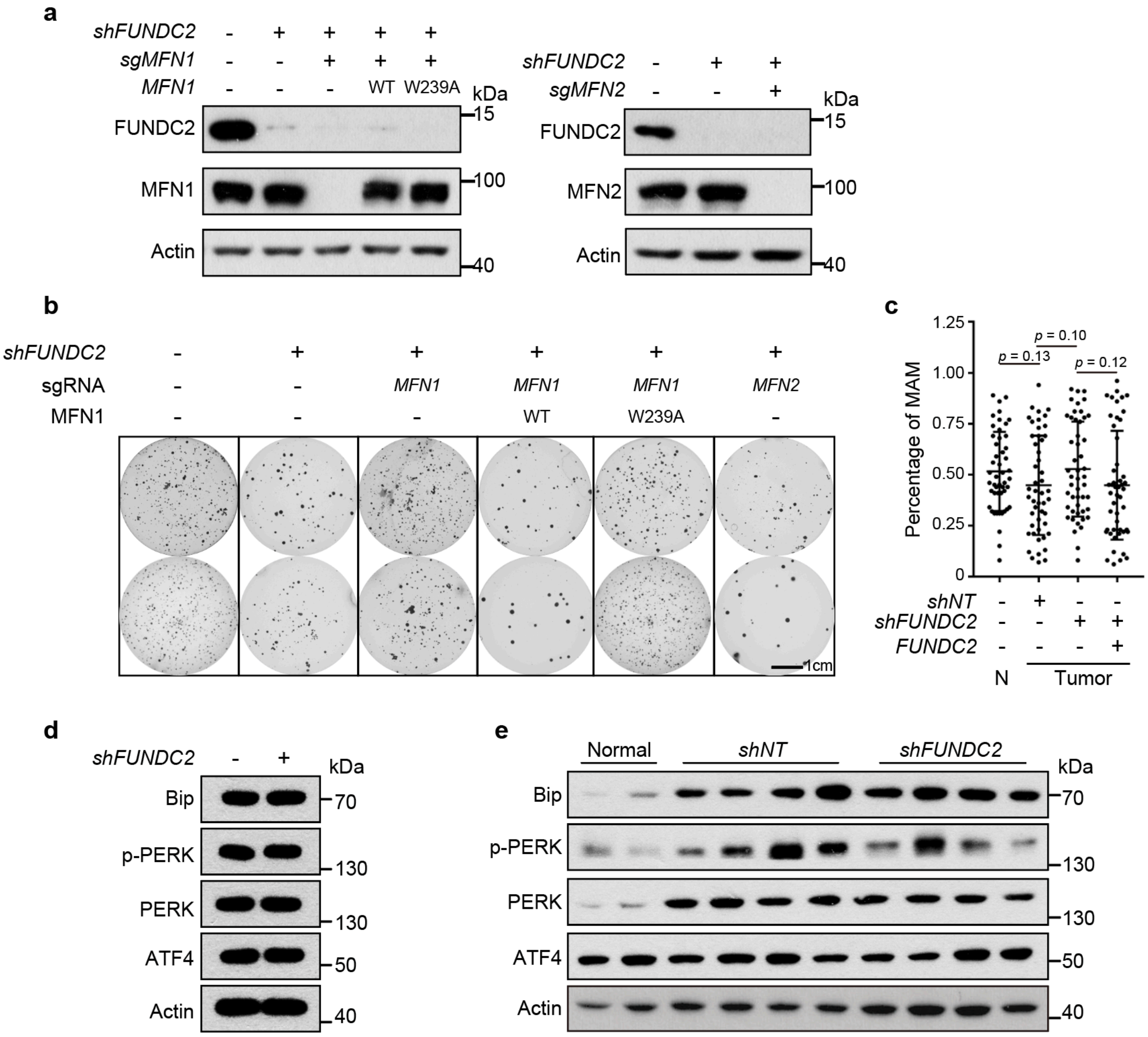

Supplementary Fig. 7 FUNDC2 does not regulate MAM. a Generation of indicated Huh-7 cell lines. Cells were infected for respective shRNAs or sgRNAs, and protein levels were determined by western blotting. **b** Representative pictures of colony formation by indicated cells. **c** Quantification of the percentage of MAM regions normalized by mitochondrial perimeter in tumors, n = 50 MAM regions. **d, e** Knockdown of *FUNDC2* does not affect ER stress. *FUNDC2* knockdown Huh-7 cells (**d**) and tumor lysates (**e**) were examined by western blotting using specific antibodies. Data is presented as mean ± SD. p values were calculated by two-tailed unpaired Student's t-test. Source data are provided as a Source Data file.

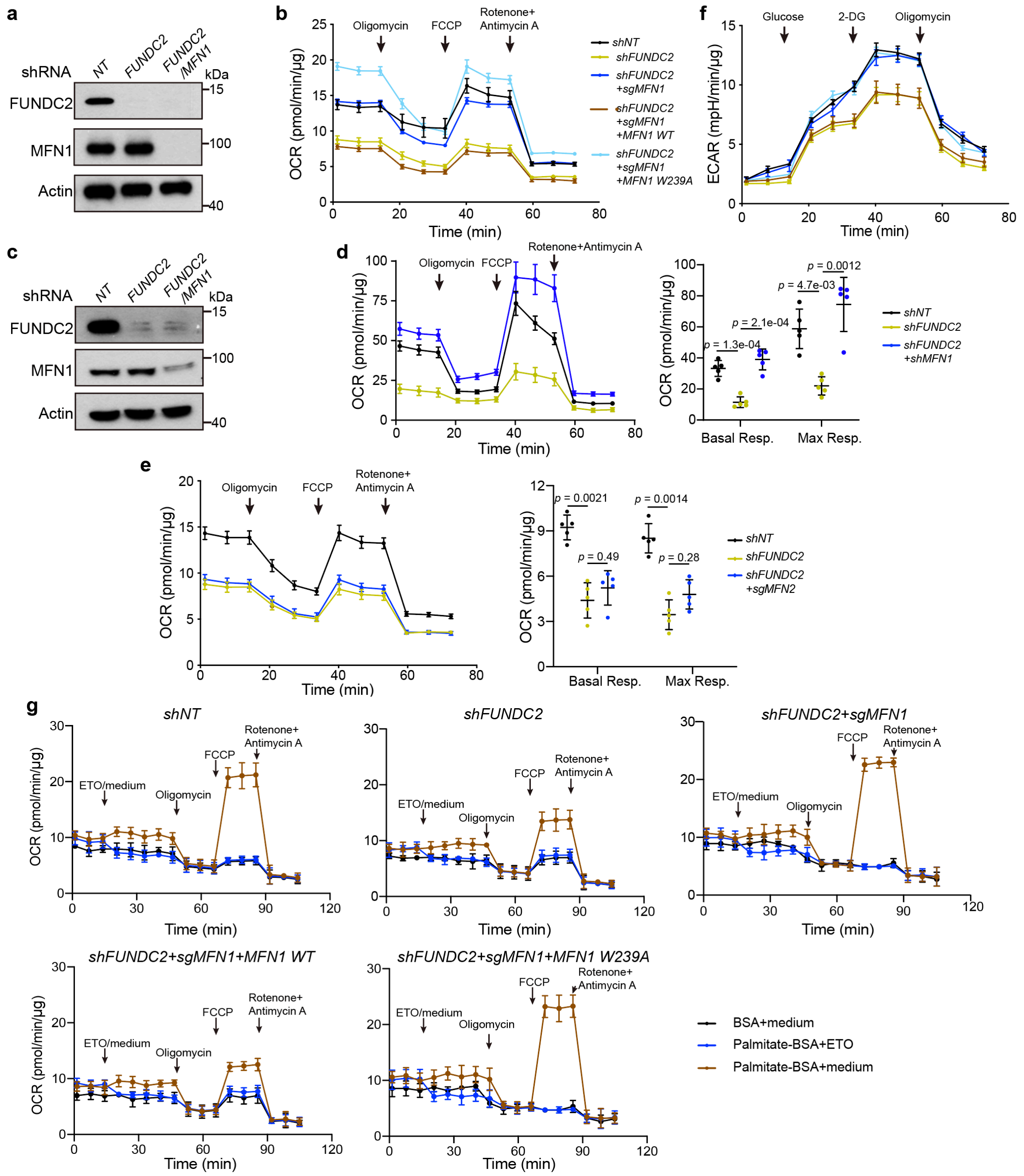

Supplementary Fig. 8 FUNDC2 regulates mitochondrial functions by inhibiting MFN1. **a** Generation of *FUNDC2* and *MFN1* double knockdown Huh-7 cells. Protein levels were determined by western blotting. **b** Seahorse analysis of OCR for indicated Huh-7 cells. n = 5. **c** Generation of *FUNDC2* and *MFN1* double knockdown HepG2 cells. **d** Knockdown of *MFN1* rescued mitochondrial respiration in *FUNDC2* knockdown HepG2 cells. Indicated cells were subjected to seahorse analysis. Quantifications of basal and maximal respiration were shown on the right, n = 5. **e** Knockout of *MFN2* does not rescue mitochondrial respiration in *FUNDC2* knockdown Huh-7 cells. Experiments were similar to (**d**). **f** Seahorse analysis of ECAR for indicated Huh-7 cells, n = 5. **g** FAO of indicated Huh-7 cells was analyzed by seahorse, n = 5. n was biological replicates for all experiments. Data is presented as mean ± SD. *p* values were calculated by two-tailed unpaired Student's *t*-test. Source data are provided as a Source Data file.

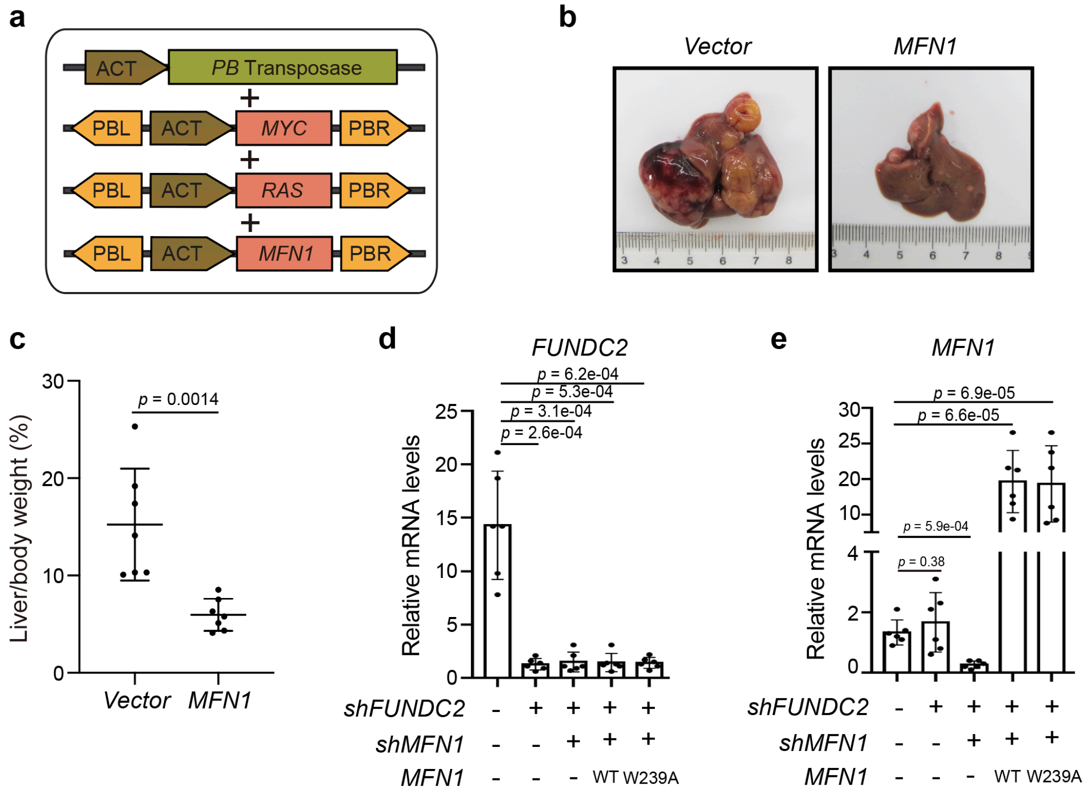

Supplementary Fig. 9 Overexpression of *MFN1* inhibits liver tumorigenesis. a Illustration of plasmids used for multiplexed genome editing *in vivo*. **b, c** Overexpression of *MFN1* suppressed tumorigenesis induced by *MYC+RAS*. Representative livers at 80 days after injection were shown (**b**). Liver/body weight ratios were quantified in (**c**), n = 7. **d, e** Determination of *FUNDC2* and *MFN1* mRNA levels by quantitative RT-PCR, n = 6. n was biological replicates for all experiments. Data is presented as mean ± SD. p values were calculated by two-tailed unpaired Student's t-test. Source data are provided as a Source Data file.

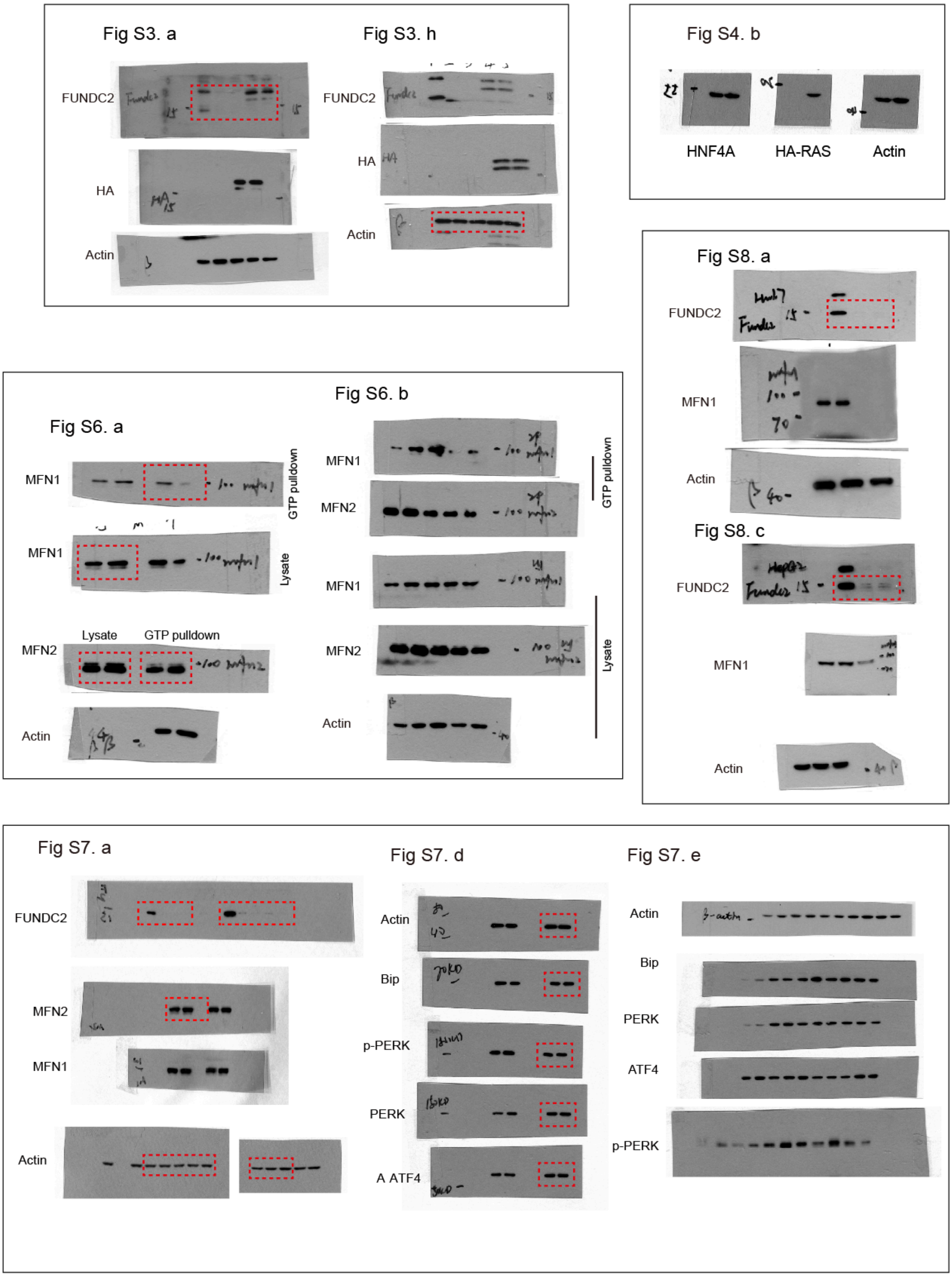

Supplementary Fig. 10 Scans of uncropped blots for supplementary figures.
