# Supplementary material for: FUNDC2 promotes liver tumorigenesis by inhibiting MFN1-mediated mitochondrial fusion
Source: Nat Commun. 2022 Jun 17;13:3486. doi: 10.1038/s41467-022-31187-6 (PMC9203792; doi:10.1038/s41467-022-31187-6)
Supplement: Supplementary file 3 — Description of Additional Supplementary Files [file 41467_2022_31187_MOESM3_ESM.pdf]

Description of Additional Supplementary Files

File Name: **Supplementary Data 1**
Description: Targeted metabolomics identified metabolites regulated by FUNDC2 in tumors. Values under sample groups represent signal intensities of each metabolite normalized by total protein concentration, n = 6 biological replicates.

File Name: **Supplementary Data 2**
Description: Reduced phopsholipids by FUNDC2 knockdown in tumors were revealed by lipidomics. Values represent signal intensities of each metabolite normalized by total protein concentration, n = 6 biological replicates.

File Name: **Supplementary Data 3**
Description: Targeted metabolomics identified metabolites regulated by FUNDC2 in an MFN1-dependent manner. Huh-7 stable cells were subjected to metabolomics analysis, n = 5 biological replicates. Values under sample groups are signal intensities of metabolites. The amount of cells used for each sample was kept the same.
