# Supplementary material for: FUNDC2 promotes liver tumorigenesis by inhibiting MFN1-mediated mitochondrial fusion
Source: Nat Commun. 2022 Jun 17;13:3486. doi: 10.1038/s41467-022-31187-6 (PMC9203792; doi:10.1038/s41467-022-31187-6)
Supplement: Supplementary file 7 — Reporting Summary [file 41467_2022_31187_MOESM7_ESM.pdf]

nature portfolio

Corresponding author(s): Bin Zhao

Last updated by author(s): May 14, 2022

Reporting Summary

Nature Portfolio wishes to improve the reproducibility of the work that we publish. This form provides structure for consistency and transparency in reporting. For further information on Nature Portfolio policies, see our Editorial Policies and the Editorial Policy Checklist.

Statistics

For all statistical analyses, confirm that the following items are present in the figure legend, table legend, main text, or Methods section.

n/a	Confirmed	
☐	☒	The exact sample size (*n*) for each experimental group/condition, given as a discrete number and unit of measurement
☐	☒	A statement on whether measurements were taken from distinct samples or whether the same sample was measured repeatedly
☐	☒	The statistical test(s) used AND whether they are one- or two-sided<br>*Only common tests should be described solely by name; describe more complex techniques in the Methods section.*
☐	☒	A description of all covariates tested
☐	☒	A description of any assumptions or corrections, such as tests of normality and adjustment for multiple comparisons
☐	☒	A full description of the statistical parameters including central tendency (e.g. means) or other basic estimates (e.g. regression coefficient) AND variation (e.g. standard deviation) or associated estimates of uncertainty (e.g. confidence intervals)
☐	☒	For null hypothesis testing, the test statistic (e.g. *F*, *t*, *r*) with confidence intervals, effect sizes, degrees of freedom and *P* value noted<br>*Give P values as exact values whenever suitable.*
☒	☐	For Bayesian analysis, information on the choice of priors and Markov chain Monte Carlo settings
☒	☐	For hierarchical and complex designs, identification of the appropriate level for tests and full reporting of outcomes
☒	☐	Estimates of effect sizes (e.g. Cohen's *d*, Pearson's *r*), indicating how they were calculated

Our web collection on statistics for biologists contains articles on many of the points above.

Software and code

Policy information about availability of computer code

Data collection	Immunofluorescence were taken by LSM 880 (Zeiss) confocal microscopy. HE or IHC stained sections were scanned with digital section scanner (KF-PRO-005) or Olympus VS120 at 10× magnification. Targeted metabolome, metabolites were quantified using triple quadrupole mass spectrometer (the QTRAP 6500+ System, ABSCIEX) coupled with an ultrahigh performance liquid chromatography. Lipidomic was performed by SCIEX QTOF 6600+ System. qPCR was performed with Hifair III One Step RT-qPCR SYBR Green Kit (YEASEN) on Bio-Rad CFX96 system. FACS was performed with Beckman CytoFLEX system and Cytexpert software (version 1.2). The Zhang laboratory CRISPR Design Tool was used to design sgRNA sequences.
Data analysis	Image J (version 1.52r), GraphPad Prism (version 6), FlowJo (version 10), SCIEX OS (version 1.7), MS-DIAL (version 4.6) , Image Pro Plus (version 6)

For manuscripts utilizing custom algorithms or software that are central to the research but not yet described in published literature, software must be made available to editors and reviewers. We strongly encourage code deposition in a community repository (e.g. GitHub). See the Nature Portfolio guidelines for submitting code & software for further information.

Data

Policy information about availability of data

All manuscripts must include a data availability statement. This statement should provide the following information, where applicable:

- Accession codes, unique identifiers, or web links for publicly available datasets
- A description of any restrictions on data availability
- For clinical datasets or third party data, please ensure that the statement adheres to our policy

All data is available in the main text or supplementary materials. Human data derived from the TCGA and GSE124535 datasets are available from https://portal.gdc.cancer.gov/legacy-archive/search/f, and https://www.ncbi.nlm.nih.gov/geo/query/acc.cgi?acc=GSE124535. Source data are provided with this paper.

Field-specific reporting

Please select the one below that is the best fit for your research. If you are not sure, read the appropriate sections before making your selection.

☒ Life sciences ☐ Behavioural & social sciences ☐ Ecological, evolutionary & environmental sciences

For a reference copy of the document with all sections, see nature.com/documents/nr-reporting-summary-flat.pdf

Life sciences study design

All studies must disclose on these points even when the disclosure is negative.

Sample size	The number of mice used in each experimental group was based on prior experience with liver cancer animal models (Genes Dev. 2017 Feb 1;31(3):247-259. doi: 10.1101/gad.294348.116; Cell. 2013 Apr 11;153(2):389-401. doi: 10.1016/j.cell.2013.03.026.). For in vivo experiments, sample size of n=7-10 was typical of experiments and was used in the study. For in vitro experiments, we used sample sizes containing two or more biological replicates which can provide adequate statistical power in biochemical analysis. All sample sizes were listed in the corresponding figure legend or figures.
Data exclusions	No data were excluded.
Replication	Animal study was repeated for two times, one month apart, considering the 3R principle. The results of all attempts were successful and consistent. All other experiments were repeated three times unless specified in figure legends. All shown results were consistent among replicates.
Randomization	Animals were randomly assigned according to standard procedure. For in vitro experiments, cells were prepared, treated, processed in a random order. The micrographs, FACS analysis were in a random order.
Blinding	For animal experiments, investigators were blinded to the group allocation during euthanizing animals, staining and assessing the outcomes. For the other experiments, investigators were not blinded to experimental groups during data collection and analysis. Data reported in this latter case is not subjective.

Reporting for specific materials, systems and methods

We require information from authors about some types of materials, experimental systems and methods used in many studies. Here, indicate whether each material, system or method listed is relevant to your study. If you are not sure if a list item applies to your research, read the appropriate section before selecting a response.

Materials & experimental systems

n/a	Involved in the study	
☐	☒	Antibodies
☐	☒	Eukaryotic cell lines
☒	☐	Palaeontology and archaeology
☐	☒	Animals and other organisms
☐	☒	Human research participants
☒	☐	Clinical data
☒	☐	Dual use research of concern

Methods

n/a	Involved in the study	
☒	☐	ChIP-seq
☐	☒	Flow cytometry
☒	☐	MRI-based neuroimaging

Antibodies

Antibodies used	Rabbit anti-FUNDC2, US Biological, Cat# 035793, application: WB; Rabbit anti-MFN1 (JF0954), HUABio, Cat#ET1702-01, application: WB, IP; Rabbit anti-MFN2, Proteintech, Cat# 12186-1-AP, application: WB, IP; Rabbit anti-TOM20 (FL-145), Santa Cruz, Cat# sc-11415, application: IF; Rabbit anti-AFP, Abcam, Cat# ab46799, application: IHC; Mouse anti-Ki67 (B56), BD, Cat# 556003, application: IHC;

	Mouse anti-HNF4a (H1415), Cosmo Bio, Cat# PPX-PP-H1415-00, application: WB; Rabbit anti-pACC S79, CST, Cat# 3661, application: WB; Rabbit anti-HA (C29F4), CST, Cat# 3724, application: WB, IP, IF; Rat anti-OLLAS (L2), Novus Biologicals, Cat#NBP1-06713, application: ICC, IF; Rabbit anti-HSP90, Proteintech, Cat# 13171-1-AP, application: WB; Mouse anti-Flag (M2), Sigma Aldrich, Cat# A8592, application: IP; Mouse anti-Myc-Tag (9B11), CST, Cat# 2276, application: WB; Rabbit anti-GFP, Abcam, Cat#6556, application: WB; Mouse anti-ACTB (7D2C10), Proteintech, Cat# 20536-1-AP, application: WB; Rabbit anti-ATF-4 (D4B8), CST, Cat# 11815, application: WB; Rabbit anti-BiP (C50B12), CST, Cat# 3177, application: WB; Rabbit anti-PERK (D11A8), CST, Cat# 5683, application: WB; Rabbit anti-Phospho-PERK (Thr980) (16F8), CST, Cat# 3179, application: WB.
Validation	Rabbit anti-FUNDC2, US Biological, we used Fundc2 knockdown Huh-7 and HepG2 cell lysate to validate this antibody in WB. Rabbit anti-MFN1, Proteintech, we used Mfn1 knockdown Huh-7 and HepG2 cell lysate, Mfn1 knockout Huh-7 cell lysate to validate this antibody in WB. The validation from manufacturer's website : Extracts from HEK293 and HeLa cells, IHC: paraffin-embedded human kidney tissue, knockdown/ knockout validation according the manufacturer's website; Rabbit anti-MFN1 (JF0954), HUABio, WB: extracts from K562 cells; Rabbit anti-MFN2, Proteintech, we used Mfn2 knockout Huh-7 cell lysate to validate this antibody in WB. The validation from manufacturer's website : WB: mouse brain and liver, rat brain and heart, IHC: paraffin-embedded human heart tissue, knockdown/ knockout validation according the manufacturer's website; Rabbit anti-TOM20 (FL-145), Santa Cruz, WB: extracts from HeLa cells, IF: HeLa cells; Rabbit anti-AFP, Abcam, WB: whole mouse liver caner lysate, IHC: paraffin-embedded human liver cancer sections and mouse embryonic Liver; Mouse anti-Ki67 (B56), BD, IHC: paraffin-embedded human liver cancer sections, FACS: human dendritic cell and monocyte; Mouse anti-HNF4a, Cosmo Bio, WB: whole human and mouse liver tissue lysate, IF: HepG2 cells, IHC: paraffin-embedded human liver and intestine sections; Rabbit anti-pACC (Ser79), CST, WB: extracts from HEK293 cells, IHC: paraffin-embedded NIH/3T3 cells and human breast carcinoma; Rabbit anti-HA (C29F4), CST, WB: extracts from HeLa cells, IHC: paraffin-embedded COS cells, IF: COS cells; Rat anti-OLLAS (L2), Novus Biologicals, WB: cell extracts expressing carboxy-terminal OLLAS-tagged protein and control cell extracts, IP: cell extracts overexpressing carboxy-terminal OLLAS-tagged protein; Rabbit anti-HSP90, Proteintech, WB: extracts from HeLa, HEK293, C6 and NIH/3T3 cells, IP: K562 cells lysate, IHC: paraffin-embedded human liver cancer; Mouse anti-Flag (M2), Sigma Aldrich, WB: extracts from HEK293 and HeLa cells, IP: extracts of HEK293 cells; Mouse anti-Myc-Tag (9B11), CST, WB: cell extracts expressing carboxy-terminal Myc-tagged protein, amino-terminal Myc-tagged protein or control cell extracts, IP: cell extracts overexpressing carboxy-terminal Myc-tagged protein or amino-terminal Myc-tagged protein, IHC: paraffin-embedded COS cell pellets, control or transfected with a carboxy-terminal Myc tagged protein, IF: COS cells transfected with a Myc-tagged protein; Rabbit anti-GFP, Abcam, WB: U2OS cell, MC3T3 cell, HeLa, HEK293 cell, IHC: mouse Tissue sections (uterus, fat, MSC, femur bone, brain, striatal neuron, medium spiny neuron), human tissue sections (Lung carcinoma cells transfected with CMV-GFP), IP: Human Cell lysate - whole cell (HEK293T cell, HeLa cell); Mouse anti-ACTB (7D2C10), Proteintech, WB: multi-cells/ tissue, HeLa cells, MCF-7 cells, HEK-293 cells, A549 cells, rice whole plant tissue, arabidopsis whole plant tissue, IHC: human colon tissue, human brain tissue, human kidney tissue, human lung tissue, human spleen tissue, human ovary tissue, human testis tissue, human placenta tissue, human heart tissue, IF: HepG2 cells; Rabbit anti-ATF-4 (D4B8), CST, WB: extracts from HEK293 and HeLa cells, IP: extracts of HEK293 cells, IF: HeLa cells; Rabbit anti-BiP (C50B12), CST, WB: extracts from A204, L929, U-87MG cells, IHC: paraffin-embedded human glioblastoma, colon carcinoma and hepatocellular carcinoma; Rabbit anti-PERK (D11A8), CST, WB: extracts from MCF7 and LN18 cells, IHC: paraffin-embedded human breast carcinoma; Rabbit anti-Phospho-PERK (Thr980) (16F8), CST, WB: extracts from AR42J cells. These antibodies were validated according to the manufacturer's website;

Eukaryotic cell lines

Policy information about cell lines

Cell line source(s)	HepG2 (ATCC, HB-8065), HEK293T (ATCC, CRL-11268), Huh-7 (JCRB cell bank, JCRB0403), HeLa (ATCC, CCL-2),
Authentication	The HEK293T, HeLa, Huh-7 and HepG2 cell line was authenticated using Short Tandem Repeat (STR) profiling at the Genetic Testing Biotechnology (Suzhou).
Mycoplasma contamination	All cell lines are negative of mycoplasma contamination.
Commonly misidentified lines<br>(See ICLAC register)	HeLa cells were used in this study. This cell was used due to its high transfection efficiency. The cell was authenticated before use.

Animals and other organisms

Policy information about studies involving animals; ARRIVE guidelines recommended for reporting animal research

Laboratory animals	Four-week-old male ICR mice were purchased from Shanghai SLAC Laboratory Animal Company. Standard laboratory chow diet for mice were purchased from XieTong Biology (Cat# 1010082), and were fed ad libitum. The SPF grade animal room was maintained humidity (45%-60%) with a 12h (7:00 a.m.-7:00 p.m.) light/dark cycle.
Wild animals	Not involved wild animals.
Field-collected samples	The study did not involve samples collected from the field.
Ethics oversight	Approved by the Zhejiang University Animal Care and Use Committee, approval number ZJU20210073.

Note that full information on the approval of the study protocol must also be provided in the manuscript.

Human research participants

Policy information about studies involving human research participants

Population characteristics	Liver cancer patients, aged 25-85, 86.8% of men and 13.2% of women.

Recruitment	Human HCC specimens were collected from the First Affiliated Hospital of Zhejiang University between 2012 to 2019, who were all diagnosed with primary HCC by pathology and underwent curative surgical resection. Written informed consent was acquired from the patients and the patients' parties. No potential self-selection bias was involved in the process of recruitment.
Ethics oversight	This study was performed in accordance with the International Ethical Guidelines for Biomedical Research Involving Human Subjects and the principles expressed in the Declaration of Helsinki, and was approved by the ethic committee of the First Affiliated Hospital of Zhejiang University.

Note that full information on the approval of the study protocol must also be provided in the manuscript.

Flow Cytometry

Plots

Confirm that:

☒ The axis labels state the marker and fluorochrome used (e.g. CD4-FITC).

☒ The axis scales are clearly visible. Include numbers along axes only for bottom left plot of group (a 'group' is an analysis of identical markers).

☒ All plots are contour plots with outliers or pseudocolor plots.

☒ A numerical value for number of cells or percentage (with statistics) is provided.

Methodology

Sample preparation	For membrane potential measurement, Huh-7 cells were incubated with Tetramethylrhodamine methyl ester perchlorate and Mitotracker for 30 min at 37°C. For mitochondria mass assay, Huh-7 cells were only stained with Mitotracker. For CMH2DCFDA staining, Huh-7 cells were resuspended and incubated with pre-warmed PBS containing CMH2DCFDA for 30 min. After the incubation, cells were washed twice with PBS and analyzed by flow cytometry.
Instrument	Beckman CytoFlex S
Software	Beckman Cytexpert (version 1.2) and FlowJo (version 10)
Cell population abundance	After the cell population is divided, the software will provide the specific number of cells in each population
Gating strategy	The main cell population is selected according to FSC/SSC parameters, and then the positive dye cell population is selected according to the negative control which does not be stained with the dye. FITC panel for CMH2DCFDA and mitotracker staining, and PE Ds Red panel for Tetramethylrhodamine methyl ester perchlorate staining.

☒ Tick this box to confirm that a figure exemplifying the gating strategy is provided in the Supplementary Information.
