# Supplementary figures and images for: FUNDC2 promotes liver tumorigenesis by inhibiting MFN1-mediated mitochondrial fusion
Source: Nat Commun. 2022 Jun 17;13:3486. doi: 10.1038/s41467-022-31187-6 (PMC9203792; doi:10.1038/s41467-022-31187-6)

f

Li\_Fig.1

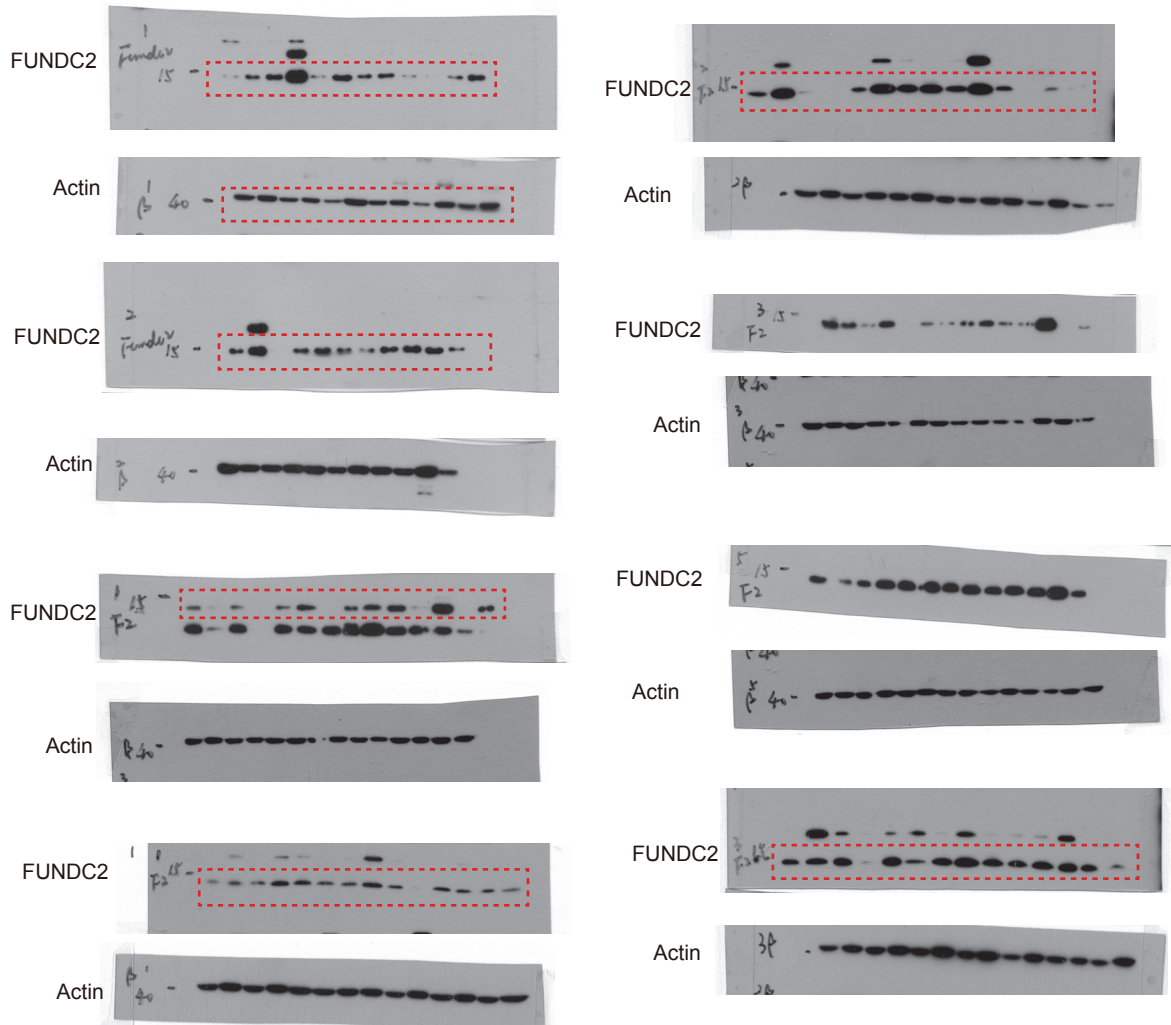

**b**

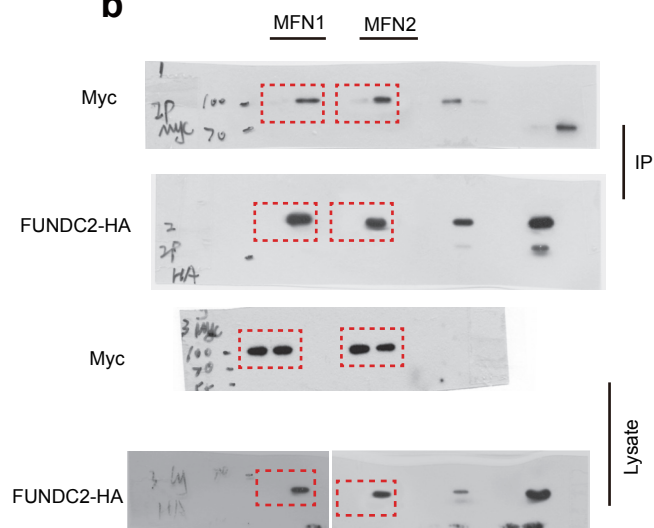

**c**

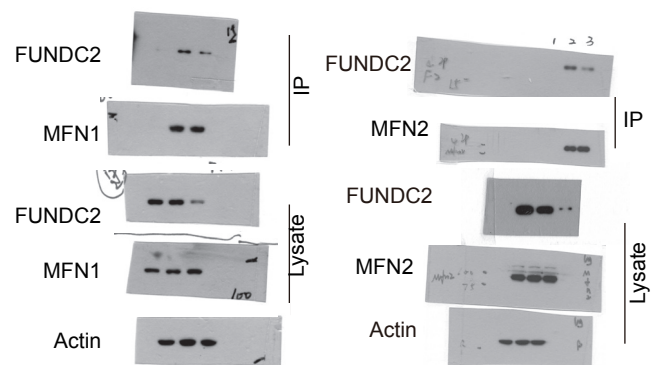

**e**

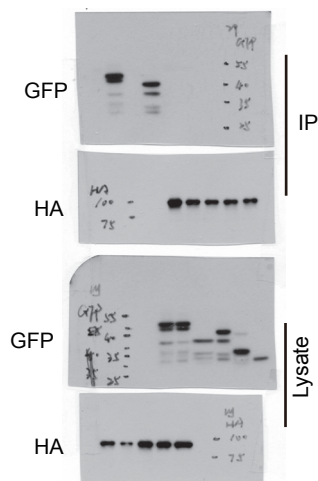

**f**

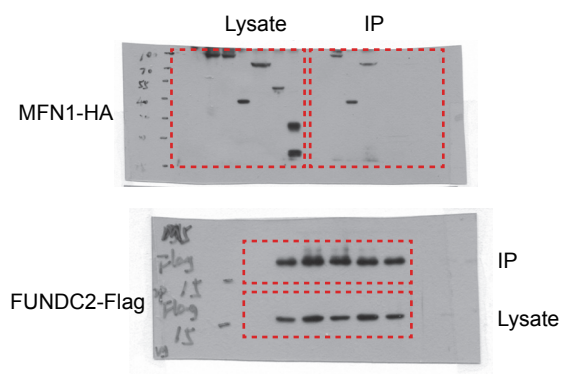

**g**

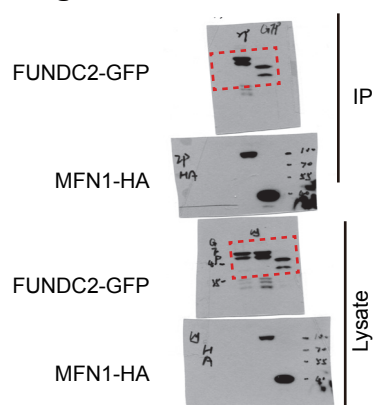

**h**

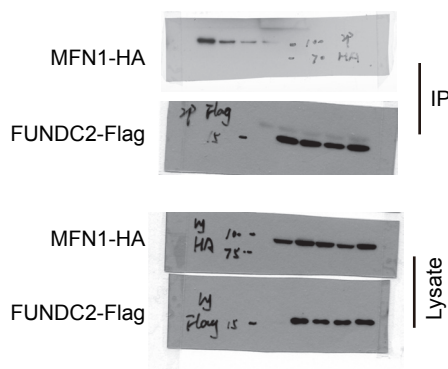

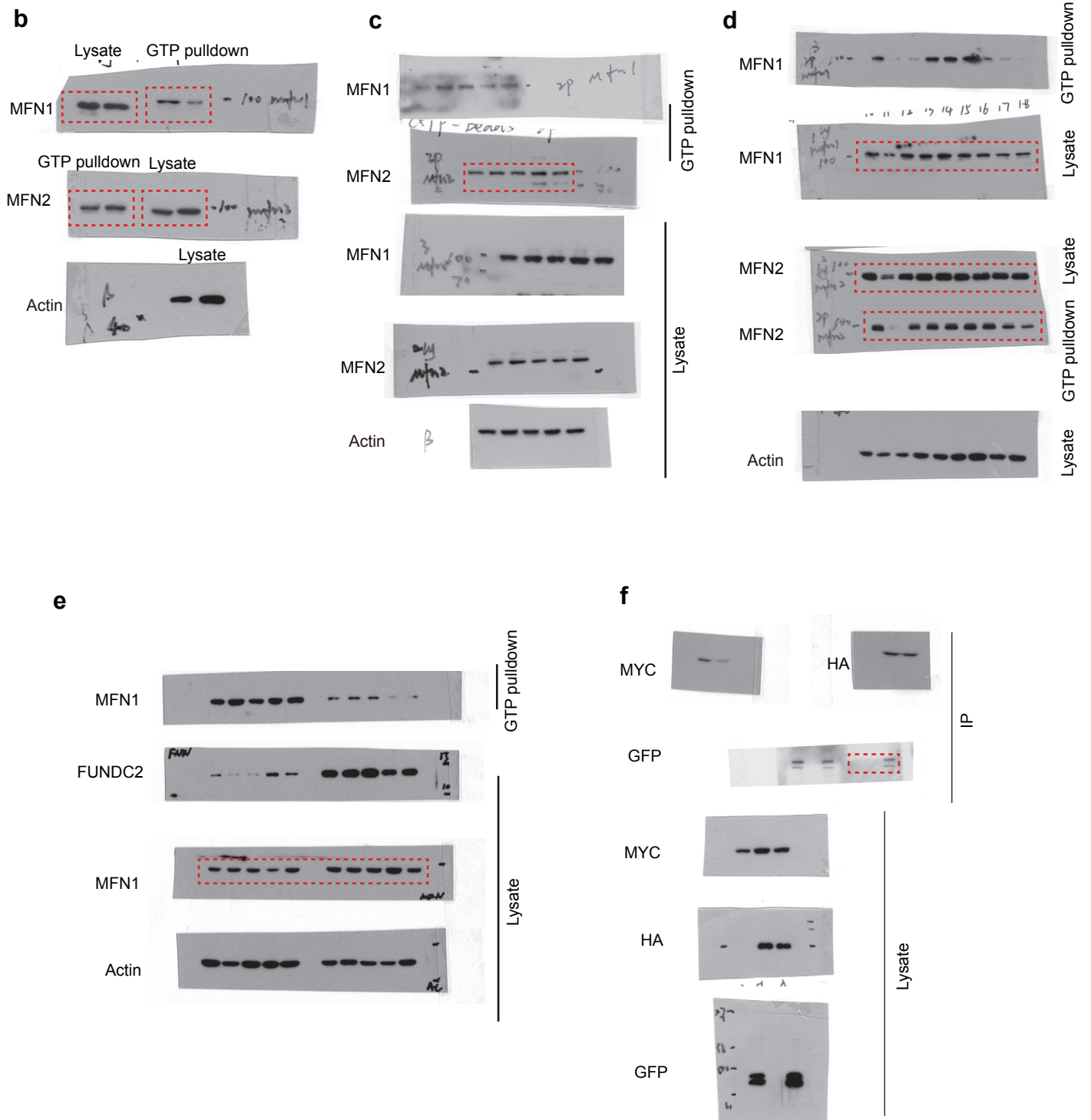

Li\_Fig.8

**g**

pACC

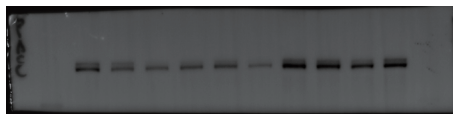

HSP90

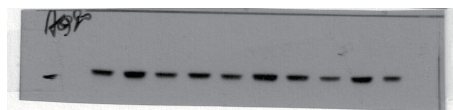

Supplement: Supplementary file 8 — Source Data [file 41467_2022_31187_MOESM8_ESM.zip › Source Data/Uncropped Blots.pdf]
